# Quality of Life of Adolescents Facing a Parental Illness: A Person-Oriented Approach

**DOI:** 10.3390/ijerph19137892

**Published:** 2022-06-27

**Authors:** Jade Pilato, Géraldine Dorard, Basilie Chevrier, Agnes Leu, Aurélie Untas

**Affiliations:** 1 Laboratoire de Psychopathologie et Processus de Santé, Université Paris Cité, 92100 Boulogne Billancourt, France; geraldine.dorard@u-paris.fr (G.D.); aurelie.untas@u-paris.fr (A.U.); 2 PSYCLE, Aix-Marseille Université, 13628 Aix-en-Provence, France; basilie.chevrier@univ-amu.fr; 3 Institute for Biomedical Ethics, University of Basel, Bernoullistrasse 28, 4056 Basel, Switzerland; agnes.leu@unibas.ch

**Keywords:** health-related quality of life, KIDSCREEN-52, adolescents, parental illness, cluster analysis, levels of caring, illness perception, academic performance

## Abstract

Studies that have investigated the health-related quality of life (HRQoL) of adolescents facing a parental illness showed inconsistent results, and none used a person-oriented approach allowing for a deeper understanding of their experience. The aim of this study was to compare the HRQoL of adolescents facing a parental illness to that of their peers, and to explore their HRQoL through a person-oriented approach. The sample consisted of 1324 adolescents recruited in secondary schools (11–15 years old). Adolescents completed questionnaires assessing sociodemographic characteristics, parental illness, HRQoL (KIDSCREEN-52), and academic performance and caring activities (MACA-YC18). Adolescents facing a parental illness showed significantly lower HRQoL than their peers on all dimensions. The cluster analysis yielded five patterns of HRQoL among adolescents facing a parental illness: *Low HRQoL*; *High HRQoL*; *Moderate HRQoL with High Social Acceptance*; *High Well-Being, High Moods and Emotions, and High Social Support and Peers*. These clusters differed according to demographics, the type of parental illness, illness perception, academic performance, and level of caring activities. The *Low HRQoL* cluster showed especially low academic performance and high level of caring activities. This multidimensional HRQoL evaluation thus helps to foreground the diversity of these adolescents’ experiences in order to better address their needs.

## 1. Introduction

Adolescence is a sensitive period of development that can be complicated by adverse life events, such as the illness of a parent. Several studies have been conducted on the psychosocial adjustment of adolescents facing a parental illness. These studies have mainly focused on oncology, although other somatic and mental illnesses have also been explored. Adolescents with a physically ill parent [1,2,3] and youth with mentally ill parents [4,5,6,7] are at greater risk of developing psychological problems. More specifically, research reported internalized problems such as depression and anxiety [3,8,9,10,11,12,13,14,15,16], and externalized problems such as delinquent or aggressive behaviors [3,8,10,12,17,18,19]. Several studies also reported physical disorders [6,20,21,22] and negative impacts on academic performance [14,15]. Moreover, these adolescents show lower self-esteem [9,13,23,24] and lower life satisfaction [21]. Research suggests that youth of parents with a mental illness are at greater risk of psychosocial maladjustment than adolescents with physically ill parents [1,25]. 

Several factors seem to be associated with adolescent adjustment, such as demographics [26,27], illness characteristics (e.g., duration, prognosis, severity, treatment), and role redistribution. Regarding illness characteristics, the literature showed inconsistent results [3,12,28,29,30,31,32,33,34]. For example, several studies found no impact of the stage or prognosis/severity of illness on children of ill parents [28,31] whereas others concluded that it did affect their adjustment [30,32,33,34]. A number of studies showed no impact of illness duration [28,29,35], whereas a meta-analysis concluded that the effects on youth adjustment were larger in studies including parents with longer illness duration [3]. However, few studies have investigated the perception of illness rather than its objective features, even though research indicated that distress would be associated with the appraisal of the seriousness and stressfulness of the illness rather than its objective characteristics [29,36,37]. In terms of role redistribution, illness often disrupts family routines and remodels the involvement of family members. As a result, adolescents may perform caring activities [21,27,31,38,39,40]. Youth can provide emotional support, render financial and practical help, and perform domestic tasks, sibling care, and personal care such as nursing-related activities or help with washing [41].

Health-related quality of life (HRQoL) is a multidimensional construct that encompasses most of the aforementioned negative effects. This non-clinical concept also considers positive aspects and is well suited to study this particular youth population. Indeed, facing a parental illness is challenging but not pathological per se [42,43]. While HRQoL is a broad concept and there is no consensus regarding its definition, it usually references the World Health Organization conceptualization of health [44], and takes into account at least physical and psychological health as well as social aspects [45]. There are tools which assess more than these three dimensions. In this regard, the KIDSCREEN [46] evaluates five (KIDSCREEN-27) to ten dimensions (KIDSCREEN-52) of children and adolescents’ HRQoL: Physical Well-Being, Psychological Well-Being, Moods and Emotions, Self-Perception, Autonomy, Relations with Parents and Home Life, Financial Resources, Social Support and Peers, School Environment, and Social Acceptance (Bullying). This metric is thus both comprehensive and versatile, providing an overall score and/or dimensional scores.

Nonetheless, few studies have investigated the HRQoL of adolescents facing a parental illness, and those that have produced conflicting results. These studies have often focused on one type of illness, conducted primarily in oncology. A Norwegian study showed that adolescents facing parental cancer have poorer HRQoL than the reference population on Physical and Emotional Well-being and on School Environment [11]. In Germany, Kühne et al. (2012) concluded that there was no difference between adolescents with a parent in the palliative stage of cancer and the normative sample, whereas Bultmann et al. (2014) showed that children of cancer survivors appeared to have a better overall HRQoL than the reference population. Another study among a Greek population showed that parent’s subjective physical health was not associated with adolescents’ HRQoL dimensions, with the exception of the Self-Perception dimension [47]. Regarding children of parents with a mental illness, research more consistently showed negative impacts on their HRQoL. Children of mentally ill parents showed lower overall HRQoL than the German reference population [7,48], and lower Physical and Psychological Well-Being [48]. Another German study concluded that maternal history of depression negatively impacts overall HRQoL, as well as the Physical Well-Being, Autonomy, and Relations with Parents and School Environment dimensions [49]. A Norwegian study including both parental mental and physical illness showed no difference between youth facing a parental illness and the reference population, except for their Physical Well-Being which was significantly poorer [42]. In addition, the authors reported no significant differences between children of parents with mental illness and those with physical illness [42].

Studies that have investigated various aspects of these adolescents’ HRQoL have explored these dimensions separately, and tested them at the level of the entire sample via a variable-oriented approach [11,47,48,49]. Yet, as parts of a holistic individual experience, there is good reason to argue that the different dimensions of HRQoL must not be considered separately. In this regard, a person-oriented approach would enable the joint exploration of various facets of HRQoL and distinguish the different configurations of HRQoL within each individual. Indeed, contrary to the variable-centered approach, the person-oriented approach consider the individual as a dynamic system [50]. This approach allows for the identification of subgroups of participants characterized by a similar experience. This perspective would thus offer invaluable help when attempting to appreciate the complexity of these adolescents’ experiences.

The aim of the present study was to determine the impact of parental illness on adolescents’ HRQoL and to explore the diversity of their experiences. The first objective was to compare the HRQoL of youth facing a parental illness with their peers. We hypothesized that adolescents having a parent affected by an illness would have a lower HRQoL than their peers. The second objective was to focus on the HRQoL of adolescents facing a parental illness through a person-oriented approach. We hypothesized that several patterns of HRQoL would emerge and be related to different characteristics, such as demographics, illness perception, and caring activities.

## 2. Method

### 2.1. Participants

This is a cross-sectional study. The sample consisted of adolescents recruited in eight secondary schools in the Paris area from January 2021 to March 2022. The inclusion criteria were to be in Year 7 to 10, to have sufficient language proficiency, to have signed the consent to participate form and to have transmitted the parental consent form. A total number of 1329 adolescents aged 11 to 15 took part in the study. Four participants were excluded from the analyses because of incomplete questionnaires and one because of an inconsistent pattern of answers. The mean age of the 1324 participants was 12.89 years (*SD* = 1.12, range 11–15); 8.84% were in Year 7, 28.78% in Year 8, 29.08% in Year 9, and 33.08% in Year 10; 56.27% were females. Concerning their health status, 8.16% reported having an illness or a disability. The adolescents’ characteristics are presented in Table 1.

### 2.2. Procedure

The study was carried out during class time. Toward this end, a short video clip providing information on the survey was played to pupils, and detailed information sheets and consent forms were handed out. The following week, those who agreed to participate and returned the signed parental consent completed a questionnaire on an electronic tablet during class-time. Completion of the questionnaires took approximately 30 min.

This research received ethics committee approval from the relevant study institutions (ethical research committee of the University Paris Cité, No. 00012020-27, registration at University for protection of personal data, No. 20200602).

### 2.3. Materials

#### 2.3.1. Sociodemographic Characteristics

A socio-demographic questionnaire collected information about age, gender, grade, foreign language spoken at home, family living arrangement, adolescent illness, presence of siblings and sibling rank.

#### 2.3.2. Parental Illness or Disability

Facing a parental illness was assessed with several questions. The adolescents had to indicate whether they had a parent with a severe or chronic illness, disability, psychological problem, substance abuse, or any other health problem and to name or explain the illness in their own words in the text box provided in the questionnaire. If so, they had to answer five questions about their perception of this illness, based on the Brief Illness Perception Questionnaire (Brief-IPQ) [51], through 4-point Likert-type scales ranging from “not at all” to “extremely”. This questionnaire evaluated the influence of the illness on their life, whether the situation affected them emotionally, the perceived duration of the illness, their perception of their parents’ difficulties due to the illness, and the extent of information they had on the illness.

#### 2.3.3. Health Related Quality of Life

The adolescent’s HRQoL was assessed with KIDSCREEN-52, validated in French [46]. This instrument evaluates the quality of life of children and adolescents between 8 and 18 years old. It is a 52-item questionnaire, which uses 5-point Likert-type scales ranging from “never” to “always” or “not at all” to “extremely”. The questions refer to the past week and measure the ten dimensions of HRQoL: Physical Well-Being (e.g., feeling fit and well), Psychological Well-Being (e.g., feeling cheerful), Moods and Emotions (e.g., feeling sad), Self-Perception (e.g., feeling jealous of the way other girls and boys look), Autonomy (e.g., having enough time to meet friends), Relations with Parents and Home Life (e.g., being happy at home), Financial Resources (e.g., having enough money to do things with friends), Social Support and Peers (e.g., being able to rely on friends), School Environment (e.g., being happy at school), Social Acceptance (Bullying) (e.g., being afraid of other girls and boys). KIDSCREEN-52 raw scores are transformed into *T*-values, with higher scores indicating higher HRQoL levels; this tool shows satisfactory psychometric properties [46].

#### 2.3.4. Perceived Academic Performance

Adolescents’ perception of their academic performance was assessed through a question (i.e., “On a scale from 0 to 10, how well do you succeed at school?) using a numerical response scale ranging from 0 (failing at school) to 10 (succeeding very well at school).

#### 2.3.5. Caregiving Activities

The Multidimensional Assessment of Caring Activities (MACA-YC 18) is a 18-item validated questionnaire used to assess the amount of caring activity undertaken by children, adolescents, and young adults between 8 and 25 years old [52]. It uses a 3-point Likert-type scale ranging from “never” to “always”. It provides a total score ranging from 0 to 36, alongside six sub-scores ranging from 0 to 6, that evaluate 6 domains: domestic tasks (e.g., cleaning other rooms), household management (e.g., taking responsibility for shopping for food), financial and practical help (e.g., helping with financial matters such as dealing with bills, banking money, collecting benefits), personal care (e.g., helping someone to have a bath or shower), emotional support (e.g., keeping someone company, for example sitting with them, reading to them, and talking to them), and sibling care (e.g., looking after brothers or sisters on one’s own). Higher scores indicate higher levels of caring activities. The questionnaire was validated in French [53].

### 2.4. Data Analysis

Descriptive analyses were performed to describe the total sample (mean, SD, frequency).

Adolescents who indicated that they had a parent with a severe or chronic illness, disability, psychological problem, substance abuse or any other health problem were considered to be facing a parental illness. Their answers to the open question in the text box on naming or explaining the health problem were recoded into two categories: physical illness (1) and mental illness (2). The first category included chronic or severe physical illness (e.g., diabetes, cancer, multiple sclerosis), physical disability (e.g., mobility impairment, sensory impairment). The second included mental illness (e.g., depression, bipolarity) and substance abuse (e.g., alcohol abuse). Minor health problems were excluded (e.g., non-severe asthma, pollen allergies) and these adolescents were not considered to be facing a parental illness.

Non-parametric tests were used because the assumption of normality was not met. A Quade’s test was performed to compare adolescents facing a parental illness to their peers on the ten KIDSCREEN-52 dimensions, controlling for gender and age, while a Mann Whitney test was used to compare these two groups on age. Chi-square analyses were then performed to compare adolescents facing a parental illness to their peers on qualitative variables such as gender, grade, foreign language spoken at home, ill/disabled adolescent, family living arrangement, having siblings, sibling rank, ill/disabled parent, and type of parental illness. Chi-square tests were only run if the expected count in each cell were greater than 5 [54].

Prior to cluster analysis, scores for the 10 dimensions of HRQoL for the subsample of adolescents facing a parental illness were standardized, and the data were inspected for missing values and multivariate outliers using a Mahalanobis distance measure. A two-step cluster analysis was then conducted. Firstly, a hierarchical cluster analysis using Ward’s method and squared Euclidean distance was run. Secondly, the cluster centers obtained in the initial step were employed as non-random starting points in an iterative *k*-means analysis. Three criteria were used to select the final number of clusters: substantive theorizing, parsimony, and explanatory power [55]. Conventional criteria were used to interpret these cluster patterns [56]: an absolute value of 0.2 *SD* defined a small effect, 0.5 *SD* a moderate effect, and 0.8 *SD* a large effect. Kruskal-Wallis and chi-square tests were performed to characterize clusters regarding age, gender, type of parental illness, illness perception, perceived academic performance and level of caring activities. Finally, post-hoc tests were conducted. Kruskal-Wallis was completed by pairwise comparisons using Mann-Whitney (U) tests with Bonferroni corrections. For chi-square tests, standardized residuals were analyzed (i.e., a value above 2 indicates a significant difference).

Analyses were performed with IBM SPSS Statistics software (version 28.0; SPSS Inc., Chicago, IL, USA) and with stats 4.1.2, ggplot2 3.3.5, rrcov 1.6–2, and MASS 7.3–55 packages in R 4.1.2 software [57].

## 3. Results

### 3.1. Identification of Adolescents Facing a Parental Illness

Among the 1324 participants, 15.71% (*n* = 208) reported a parental illness. More than one in ten (12.02%) reported that both parents were ill; 48.56% had an ill mother and 39.42% an ill father. About 76% of ill parents had a chronic/severe physical illness or disability, and 24.04% had a mental illness including substance abuse. The characteristics are presented in Table 1.

### 3.2. Comparison between Adolescents Facing a Parental Illness and Adolescents with Healthy Parents

Adolescents facing a parental illness were more likely to be girls (*p* < 0.01) compared to adolescents with healthy parents. They were less likely to be the oldest sibling (*p* < 0.05) and more likely to have a health problem themselves (*p* < 0.001) compared to adolescents with healthy parents (see Table 1). No other difference was found between adolescents facing a parental illness and their peers regarding sociodemographic characteristics (i.e., age, grade, foreign language spoken at home, type of adolescent’s illness, family living arrangement, having siblings). Adolescents facing a parental illness had significantly lower HRQoL than their peers on the ten KIDSCREEN-52 dimensions (see Table 1).

### 3.3. Patterns of HRQoL among Adolescents Facing a Parental Illness

Combining the ten dimensions of HRQoL, the analysis yielded a five-cluster solution. This solution accounted for 43.10% of the variance in Physical Well-Being, 60.70% in Psychological Well-Being, 53.48% in Moods and Emotions, 41.59% in Self-Perception, 50.04% in Autonomy, 55.45% in Relations with Parents and Home Life, 26.34% in Financial Resources, 34.76% in Social Support and Peers, 39.21% in School Environment, and 49.55% in Social Acceptance. A discriminant function analysis supported this final cluster solution: Wilks’ lambda = 0.054, χ^2^ (40) = 580.62, *p* < 0.001; 92.31% of cross-validated grouped cases correctly classified.

**Figure 1:**
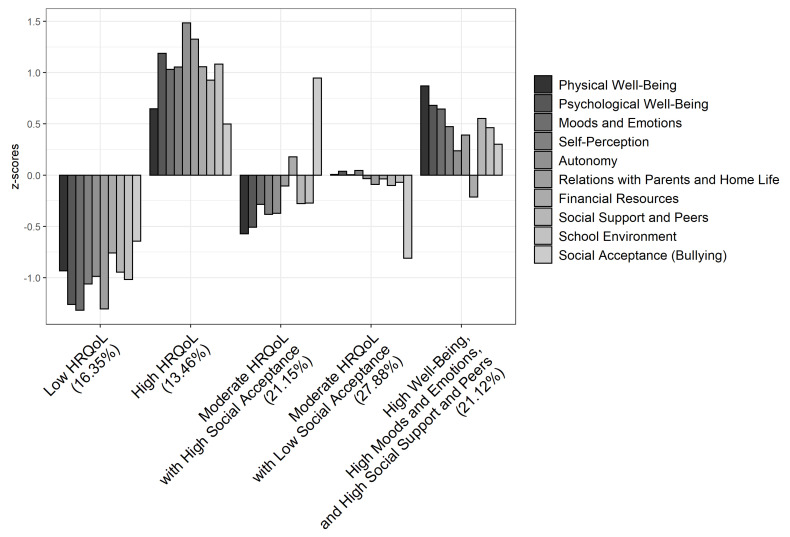
Cluster solution for HRQoL. *n* = 208. z-scores for Physical Well-Being, Psychological Well-Being, Moods and Emotions, Self-Perception, Autonomy, Relations with Parents and Home Life, Financial Resources, Social Support and Peers, School Environment, Social Acceptance.

The *Low HRQoL* cluster scored low to very low on all dimensions, and represented 16.35% of the sample. Scores were especially low on Psychological Well-Being, Moods and Emotions, Parents and Home Life dimensions. In parallel fashion, the *High HRQoL* cluster, representing 13.46% of the sample, scored high to very high on all dimensions. Adolescents in the *High HRQoL* cluster showed very high scores on Psychological Well-Being, Autonomy, Parents and Home Life dimensions. The *Moderate HRQoL with High Social Acceptance* cluster represented 21.15% of the sample, and was characterized by low scores on Physical and Psychological Well-Being dimensions, a high score on the Social Acceptance dimension, and intermediate scores on other dimensions. The *Moderate HRQoL with Low Social Acceptance* cluster scored low on the Social Acceptance dimension and intermediate in all other dimensions; about 28% of participants presented this HRQoL pattern. *The High Well-Being, High Moods and Emotions, and High Social Support and Peers* cluster scored high on Physical and Psychological Well-Being, Moods and Emotions, Social Support and Peers dimensions and intermediate to moderately high in other domains. About 21% of adolescents belonged to this cluster (see Figure 1).

### 3.4. Patterns of HRQoL Characterization

#### 3.4.1. Demographics

Clusters significantly differed on age (*p* < 0.001), gender (*p* < 0.001) and type of parental illness (*p* < 0.001). Post-hoc tests revealed that adolescents were younger in the *High HRQoL* and in the *High Well-Being, High Moods and Emotions, and High Social Support and Peers* clusters compared with the *Moderate HRQoL with High Social Acceptance* cluster. Males were overrepresented in the *High HRQoL* and the *High Well-Being, High Moods and Emotions, and High Social Support and Peers* clusters. Regarding the type of parental illness, mental illness was overrepresented in the *Low HRQoL* cluster and underrepresented in the *High Well-Being, High Moods and Emotions, and High Social Support and Peers* cluster. Additional characteristics are presented in Table 2. No significant differences were found for the other characteristics (i.e., foreign language spoken at home, ill/disabled adolescent, family living arrangement, sibling rank, ill/disabled parent). No conclusion could be drawn regarding the type of adolescents’ illness and whether they had siblings.

#### 3.4.2. Perceived Academic Performance, Illness Perception, and Caring Activities

The results showed significant differences between clusters regarding perceived academic performance (*p* < 0.001), the reported impact of the parental illness on adolescents’ lives (*p* < 0.05), and adolescents’ perception of their parents’ difficulties due to the illness (*p* < 0.05). According to post-hoc tests, the *Low HRQoL* cluster scored lower than the other four clusters on perceived academic performance, whereas the *High HRQoL* cluster scored higher than the *Moderate HRQoL with Low Social Acceptance* cluster. Post-hoc tests also showed that adolescents in the *Low HRQoL* pattern reported more parental difficulties due to the illness and more impact on their life than those in the *High HRQoL* cluster (see Table 2).

There were significant differences between clusters regarding total MACA score (*p* < 0.001), financial/practical care (*p* < 0.05), personal care (*p* < 0.01), emotional care (*p* < 0.01), and sibling care scores (*p* < 0.01). On the total MACA score, post-hoc tests showed that the *Low HRQoL* cluster scored higher than the *High HRQoL* and the *Moderate HRQoL with High Social Acceptance* clusters. The *High HRQoL* cluster scored lower than the *Moderate HRQoL with Low Social Acceptance* and the *High Well-Being, High Moods and Emotions, and High Social Support and Peers* clusters. Moreover, the *High Well-Being, High Moods and Emotions, and High Social Support and Peers* cluster scored higher than the *Moderate HRQoL with High Social Acceptance* cluster (see Table 2). Regarding sub-scales, post-hoc tests revealed that the *Low HRQoL* cluster scored higher than the *High HRQoL* cluster on financial/practical care and emotional care. The *Low HRQoL* cluster scored higher than the *High HRQoL* and the *Moderate HRQoL with High Social Acceptance* cluster on personal care. The *High Well-Being, High Moods and Emotions, and High Social Support and Peers* cluster scored higher than the *Moderate HRQoL with High Social Acceptance* cluster on sibling care (see Table 2).

## 4. Discussion

This was the first population-based study to explore the HRQoL of adolescents facing a parental illness through a person-oriented approach employing a multidimensional conceptualization of HRQoL. The inclusion of a large sample of adolescents in the general population enabled the ecological construction of groups, without *a priori* selection of the type of parental illness. It also enabled a comparison of the characteristics of adolescents confronted with a parental illness to a control group made up of their peers. This person-oriented approach allowed for a better understanding of the diversity and complexity of these youths’ experiences. Adolescents facing a parental illness have a lower HRQoL than their peers on all dimensions. Nonetheless, adolescents facing a parental illness do not experience the situation the same way, and some adjust better than others do. Their HRQoL differed according to demographics, perceived academic performance, type of parental illness, illness perception, and levels of caring activities.

The comparative analyses showed that adolescents who faced a parental illness had lower HRQoL scores than their peers who did not report experiencing a parental illness. This finding is consistent with previous studies in oncology [11] and with children of parents with mental illness [34,48,49]. Our study is the first to report results on such a wide variety of dimensions of health-related quality of life. Indeed, few studies have investigated dimensions such as Social Acceptance and Financial Resources. This holistic investigation showed that all domains of adolescents’ HRQoL were impaired in the group of adolescents facing a parental illness. In addition to poorer HRQoL, adolescents facing a parental illness were more likely to have an illness themselves compared with their peers. It could be due to the heritability of some illnesses [10,58] and to the distress caused by the illness resulting in mental health problems [11,14,16,17] or somatization [21].

Nonetheless, adolescents facing a parental illness cannot be regarded as a homogeneous group. The cluster analysis provided never-before-seen insight into this heterogeneity, yielding five patterns of HRQoL among adolescents facing a parental illness. The *Low HRQoL* cluster, scoring low to very low across all ten dimensions, was quite distinct from the other four clusters and clearly differed on several variables, diverging most sharply from the *High HRQoL* cluster displaying the best HRQoL.

Adolescents in the *Low HRQoL* cluster were more likely to be facing mental illness, which is consistent with literature showing that adolescents of parents with mental illness are at higher risk of psychosocial maladjustment than youth with physically ill parents [1,25]. This could be explained by the fact that youth of parents with mental illness live an unpredictable everyday life [59]. Indeed, mental illness can be associated with unstable parental behaviors [59], as well as impaired child-parent interactions and parenting practices [60]. Previous studies also showed that impaired parental mental health negatively impacted youth along several important dimensions, including Physical Well-being [47,48,49], Psychological Well-Being [47,48], Moods and Emotions [47], Parent-Child Relation, and School Environment [47,49]. Extending these findings, our study provides additional insights into the impacts of parental mental illness, as it also reveals negative effects on Self-Perception, Autonomy, Financial Resources, Social Support, and Peers and Social Acceptance (Bullying).

Adolescents in the *Low HRQoL* cluster reported that their parents had significantly more difficulties associated with their illness, and that the illness had more influence on their life than in the *High HRQoL* cluster. Our results are thus in line with literature showing that adolescents’ distress is associated with the perception of illness [29,36]. In light of these results, providing information about parental illness might help these adolescents to better cope with the situation [58]. In fact, several studies have shown positive effects of psycho-education interventions on children’s internalized and externalized symptoms [61].

Adolescents in the *Low HRQoL* cluster showed significantly higher propensity to perform caring activities than those in the *High HRQoL* cluster and in the *Moderate HRQoL with High Social Acceptance*. Therefore, low quality of life seems to be associated with high levels of caring activities. This is in line with a previous study showing that HRQoL of children of ill parents was negatively associated with the responsibilities attached to the illness [62]. Moreover, caring seem to affect every dimension of HRQoL. This is consistent with studies showing that caring influences various aspects of adolescents’ lives; research has shown major detrimental effects on their physical [63,64] and psychological well-being [63], as well as their school [65,66] and social life [66,67]. More specifically, the *Low HRQoL* cluster performed more financial/practical care and emotional care than the *High HRQoL* cluster, and more personal care than the *High HRQoL* cluster and the *Moderate HRQoL with High Social Acceptance* clusters. Financial/practical care, personal care, and emotional care thus seem to be the caring activities that exhibit the strongest associations with HRQoL as a whole. Previous studies demonstrated that personal intimate care was particularly demanding and difficult [68,69], as well as associated with negative outcomes [70,71]. This was also the case for emotional care [68,70], which has itself been previously negatively associated with HRQoL [62].

Finally, adolescents in the *Low HRQoL* cluster were more likely to show low perceived academic performance. Indeed, they reported significantly lower perceived academic performance compared to the other clusters. As every aspect of their HRQoL is impaired, this group of adolescents may have trouble finding resources to rely upon and help them to deal with their schoolwork. Additionally, the poor perceived academic performance could be explained by the group’s high level of caring tasks; indeed, the literature has shown that caring responsibilities could lead to poor grades and school achievement, as these adolescents have less time to dedicate to their school work and more difficulties concentrating in class [65,66,72].

After the *High HRQoL* cluster, adolescents in the *High Well-Being, High Moods and Emotions, and High Social Support and Peers* cluster have the highest level of HRQoL. As with the *High HRQoL* cluster, adolescents in the *High Well-Being, High Moods and Emotions, and High Social Support and Peers* cluster were younger than in *the Moderate HRQoL with High Social Acceptance* cluster and more likely to be boys. This is consistent with the literature showing that boys report higher HRQoL than girls [26,47,73], as well as previous research showing that younger youth report higher HRQoL [26,47,73]. Adolescents in the *High Well-Being, High Moods and Emotions, and High Social Support and Peers* cluster showed significantly higher caring activities than in the *Moderate HRQoL with High Social Acceptance* cluster. This difference can be accounted for by sibling care, as these two clusters differed on this dimension. Contrary to financial/practical care, personal care, and emotional care, sibling care does not seem to be negatively associated with adolescents’ HRQoL.

An additional notable finding was that the Social Acceptance dimension stood out in the cluster analysis, with two clusters differing on this dimension. The *Moderate HRQoL with High Social Acceptance* cluster, in particular, displayed several unique characteristics. First, this cluster had a higher number of girls; therefore, girls seem less likely to be victims of bullying. This is consistent with literature showing that girls report higher level of social acceptance than boys [47,74]. Additionally, having low Social Acceptance seems to be associated with both poor perceived academic performance and high levels of caring activities. Indeed, *the Moderate HRQoL with Low Social Acceptance* cluster scored significantly lower than the *High HRQoL* cluster on perceived academic performance. This suggests that victims of bullying may be at risk of low school achievement. It follows that bullying seems an important aspect to consider, and yet few studies took this dimension of HRQoL into account. Moreover, adolescents in the *Moderate HRQoL with Low Social Acceptance* cluster reported higher caring activities compared to the *High HRQoL* cluster. This suggests a possible association between caring and bullying. In addition to stigmatization due to the illness [59,66,75,76], bullying could be accounted for by their caring role. This is in line with previous research indicating that adolescents who care for a relative are more prone to be bullied at school because of their caring role [65,66,77,78].

### Limitations and Perspectives

This study has several limitations. First, our sample size of adolescents facing a parental illness was rather small (*n* = 208), and did not allow for interpretation of some analyses. Second, the identification of parental illness is based solely on adolescents’ own reports, raising the possibility that illnesses might have been underreported. Some adolescents may not reveal that they are facing a parental illness because they fear stigma or because they feel this information it too intimate. Moreover, they may not be aware that their parents suffer from a physical or mental illness, as parents might be reluctant to share this information with their children. Third, a subjective measure was used for perceived academic performance, which may be biased and not account for their actual academic achievement. Fourth, adolescents were included during the COVID-19 crisis, introducing the possibility that hardships stemming from the pandemic may have biased their HRQoL report.

Further research should be conducted on larger samples of adolescents facing a parental illness and include an objective measure of perceived academic performance. Moreover, future research should use a multicenter approach to more precisely identify adolescents facing a parental illness. In our study, males reported fewer parental illnesses and younger adolescents fewer mental illnesses; it would thus be interesting and informative to explore the identification of parental illness by adolescents in association with such developmental aspects. Also, as social acceptance seems to be an important element, future studies should explore bullying among adolescents facing a parental illness. Finally, the present study highlighted the importance of caring activities and their association with HRQoL. It follows that future research should investigate the HRQoL of Young Carers, the children and adolescents who provide or intend to provide significant or substantial care, assistance, or support to another family member who has an illness or a disability [79].

## 5. Conclusions

This is the first study to explore the health-related quality of life (HRQoL) of adolescents facing a parental illness using a person-oriented approach. Adolescents facing a parental illness showed lower HRQoL than their peers. Nonetheless, this group of adolescents cannot be regarded as a homogeneous group. Some of these adolescents seemed more impacted by the situation than others and showed low to very low HRQoL in all domains. These youth more often had a parent with a mental illness and performed high level of caring activities. As this high-risk group seems more likely to endorse a caring role, future research should focus on Young Carers and more deeply investigate the impact of caring roles on adolescents’ HRQoL.

## Figures and Tables

**Table 1:** Characteristics of the Total Sample and Characteristics of Adolescents with Healthy Parents compared with Adolescents Facing a Parental Illness.

	Total Sample *n* = 1324	Adolescents with Healthy Parents *n* = 1116	Adolescents Facing a Parental Illness *n* = 208	*U*/χ^2^	*df*	*p*
Gender, *n* (%, *ASR*)				4.38	1	*
Female	745 (56.27)	617 (55.29, −**2.09**)	128 (61.54, **2.09**)			
Male	563 (42.52)	490 (43.91, **2.09**)	73 (35.10, −**2.09**)			
Age, *M* (*SD*)	12.89 (1.12)	12.86 (1.13)	13.00 (1.08)	1.89	-	0.06
Grade, *n* (%, *ASR*)				4.15	3	0.25
Year 7	117 (8.84)	100 (8.96, 0.38)	17 (8.17, −0.38)			
Year 8	381 (28.78)	332 (29.75, 1.83)	49 (23.56, −1.83)			
Year 9	385 (29.08)	321 (28.76, −0.56)	64 (30.77, 0.56)			
Year 10	438 (33.08)	360 (32.26, −1.45)	78 (37.50, 1.45)			
Foreign language spoken at home, *n* (%, *ASR*)	428 (32.33)	361 (32.35, 0.04)	67 (32.21, −0.04)	0.00	1	0.97
Ill/disabled adolescent, *n* (%, *ASR*)	108 (8.16)	77 (6.90, −**3.87**)	31 (14.90, **3.87**)	14.99	1	***
Type of adolescent’s illness, *n* (%, *ASR*)				3.71	1	0.05
Physical illness	47 (3.55)	38 (3.41, 1.93)	9 (4.33, −1.93)			
Mental illness	61 (4.61)	39 (3.49, −1.93)	22 (10.58, 1.93)			
Family living arrangement, *n* (%, *ASR*)				3.71	1	0.05
With both parents	900 (67.98)	771 (69.09, 1.93)	129 (62.02, −1.93)			
With parents separately or one parent	405 (30.59)	330 (29.57, −1.93)	75 (36.06, 1.93)			
Having siblings, *n* (%, *ASR*)	1248 (94.26)	1055 (94.53, 0.99)	193 (92.79, −0.99)	0.99	1	0.32
Sibling rank, *n* (%, *ASR*)				7.39	2	*
The oldest	464 (35.05)	409 (36.65, **2.71**)	55 (26.44, −**2.71**)			
The youngest	453 (34.21)	374 (33.51, −1,46)	79 (37.98, 1,46)			
Middle	331 (25.00)	272 (24.37, −1.39)	59 (28.37, 1.39)			
Ill/disabled parent, *n* (%)	208 (15.71)	-		-	-	-
Mother	101 (7.63)	-	101 (48.56)			
Father	82 (6.19)	-	82 (39.42)			
Both	25 (1.89)	-	25 (12.02)			
Type of parental illness, *n* (%)				-	-	-
Physical illness	158 (11.93)	-	158 (75.96)			
Mental illness	50 (3.78)	-	50 (24.04)			
HRQoL, *M* (*SD*)						
Physical Well-Being	44.62 (9.94)	44.99 (9.83)	42.62 (10.31)	10.22	1	**
Psychological Well-Being	44.49 (10.58)	45.19 (10.58)	40.71 (9.80)	25.71	1	***
Moods and Emotions	49.55 (12.13)	50.49 (11.92)	44.50 (12.07)	35.11	1	***
Self-Perception	47.97 (10.14)	48.75 (10.13)	43.80 (9.11)	38.53	1	***
Autonomy	44.60 (10.60)	45.22 (10.65)	41.28 (9.69)	26.38	1	***
Relations with Parents and Home Life	45.40 (11.03)	46.23 (10.99)	40.94 (10.11)	39.17	1	***
Financial Resources	45.98 (11.48)	46.26 (11.38)	44.46 (11.90)	5.10	1	*
Social Support and Peers	46.80 (10.87)	47.19 (10.79)	44.68 (11.06)	10.50	1	**
School Environment	46.94 (9.54)	47.28 (9.60)	45.11 (9.04)	4.77	1	*
Social Acceptance	50.95 (9.47)	51.45 (9.31)	48.28 (9.87)	21.49	1	***

Note. For gender, 16 participants indicating another answer were excluded from analyses. For family living arrangement, 19 participants indicating another arrangement were excluded from analyses. ASR = adjusted standardized residuals. Bold ASR reflects a significant over- or underrepresentation. * *p* < 0.05. ** *p* < 0.01. *** *p* < 0.001.

**Table 2:** HRQoL Cluster Characteristics.

	HRQoL Clusters					χ^2^ (*df*)	*p*	Post-Hoc Comparisons
	Cluster 1 *n* = 34	Cluster 2 *n* = 28	Cluster 3 *n* = 44	Cluster 4 *n* = 58	Cluster 5 *n* = 44			
Age, *M (SD)*	13.18 (1.03)	12.71 (1.05)	13.45 (1.02)	12.88 (1.09)	12.77 (1.08)	14.06 (4)	**	3 > 2; 3 > 5
Gender, *n* (%, *ASR*)						33.52 (4)	***	-
Female	22 (64.71, 1.77)	12 (42.86, −**2.47**)	39 (88.64, **4.15**)	38 (65.52, 0.34)	17 (38.64, −**3.91**)			
Male	6 (17.65, −1.77)	16 (57.14, **2.47**)	4 (9.09, −**4.15**)	20 (34.48, −0.34)	27 (61.36, **3.91**)			
Foreign language spoken at home, *n* (%, *ASR*)	18 (52.94, **2.83**)	7 (25.00, −0.88)	14 (31.82, −0.06)	15 (25.86, −1.22)	13 (29.55, −0.43)	8.58 (4)	0.07	-
Ill/disabled adolescent, *n* (%, *ASR*)	8 (23.53, 1.54)	1 (3.57, −1.81)	7 (15.91, 0.21)	10 (17.24, 0.59)	5 (11.36, −0.74)	5.55 (4)	0.23	-
Type of adolescent’s illness, *n* (%, *ASR*)								
Physical illness	1 (2.94, −1.20)	1 (3.57, 1.59)	1 (2.27, −0.98)	2 (3.45, −0.76)	4 (9.09, **2.74**)	-	-	-
Mental illness	7 (20.59, 1.20)	0 (0.00, −1.59)	6 (13.64, 0.98)	8 (13.79, 0.76)	1 (2.27, −**2.74**)	-	-	-
Family living arrangement, *n* (%, *ASR*)						7.57 (4)	0.11	-
With both parents	22 (64.71, 0.45)	17 (60.71, −0.03)	23 (52.27, −1.49)	33 (56.90, −1.18)	34 (77.27, **2.42**)			
With parents separately or one parent	11 (32.35, −0.45)	10 (35.71, 0.03)	20 (45.45, 1.49)	25 (43.10, 1.18)	9 (20.45, −**2.42**)			
Having siblings, *n* (%, *ASR*)	30 (88.24, −1.12)	25 (89.29, −0.77)	40 (90.91, −0.54)	54 (93.10, 0.11)	44 (100.00, 2.08)	-	-	-
Sibling rank, *n* (%, *ASR*)						7.94 (8)	0.44	-
The oldest	8 (23.53, −0.24)	3 (10.71, −1.96)	9 (20.45, −0.94)	18 (31.03, 0.93)	17 (38.64, 1.70)			
The youngest	12 (35.29, −0.11)	14 (50.00, 1.64)	16 (36.36, −0.13)	21 (36.21, −0.36)	16 (36.36, −0.70)			
Middle	10 (29.41, 0.36)	8 (28.57, 0.17)	15 (34.09, 1.07)	15 (25.86, −0.52)	11 (25.00, −0.91)			
Type of parental illness, *n* (%, *ASR*)						19.81 (4)	***	-
Physical illness	19 (55.88, −**3,00**)	25 (89.29, 1.77)	29 (65.91, −1.76)	44 (75.86, −0.02)	41 (93.18, **3.01**)			
Mental illness	15 (44.12, **3.00**)	3 (10.71, −1.77)	15 (34.09, 1.76)	14 (24.14, 0.02)	3 (6.82, −**3.01**)			
Ill/disabled parent								-
Mother, *n* (%, *ASR*)	21 (61.76, 1.68)	14 (50.00, 0.16)	18 (40.91, −1.14)	26 (44.83, −0.67)	22 (50.00, 0.22)	3.79 (4)	0.44	
Father, *n* (%, *ASR*)	10 (29.41, −1.31)	10 (35.71, −0.43)	20 (45.45, 0.92)	24 (41.38, 0.36)	18 (40.91, 0.23)	2.39 (4)	0.66	
Both, *n* (%, *ASR*)	3 (8.82, −0.63)	4 (14.29, 0.40)	6 (13.64, 0.37)	8 (13.79, 0.49)	4 (9.09, −0.67)	-	-	
Perceived academic performance, *M (SD)*	4.94 (2.39)	7.79 (2.11)	7.30 (1.59)	6.58 (1.95)	7.45 (1.39)	37.71 (4)	***	1 < 2; 1 < 3; 1 < 4; 1 < 5; 4 < 2
Illness perception, *M (SD)*								
Illness influence	1.35 (1.15)	0.50 (0.75)	1.16 (1.08)	0.96 (1.02)	0.89 (0.87)	11.93 (4)	*	1 > 2
Perceived duration	2.18 (0.97)	1.89 (1.20)	2.02 (1.00)	2.26 (0.88)	2.02 (1.07)	2.60 (4)	0.63	
Perceived difficulties	1.44 (0.99)	0.68 (0.95)	1.14 (1.07)	1.26 (1.04)	1.07 (1.00)	10.27 (4)	*	1 > 2
Information about the problem	0.88 (0.88)	1.54 (0.96)	1.36 (1.16)	1.23 (0.98)	1.43 (1.09)	8.00 (4)	0.09	
Emotional impact	1.59 (1.26)	1.07 (0.98)	1.43 (1.21)	1.25 (1.09)	1.41 (1.06)	3.19 (4)	0.527	
Caring activities, *M (SD)*								
MACA global score	15.06 (6.35)	8.93 (4.27)	9.86 (4.02)	12.35 (4.39)	12.89 (4.98)	28.59 (4)	***	1 > 2; 1 > 3; 4 > 2; 5 > 2; 5 > 3
Domestic tasks	3.88 (1.15)	3.21 (1.34)	3.57 (1.09)	3.61 (1.21)	3.86 (1.17)	7.72 (4)	0.10	
Household management	2.94 (1.30)	2.46 (1.32)	2.25 (1.33)	2.68 (1.14)	2.86 (1.31)	7.35 (4)	0.12	
Financial/practical care	1.00 (1.39)	0.14 (0.36)	0.39 (0.75)	0.49 (0.85)	0.45 (0.90)	10.74 (4)	*	1 > 2
Personal care	1.59 (2.13)	0.32 (0.91)	0.30 (0.73)	0.79 (1.46)	0.70 (1.52)	13.99 (4)	**	1 > 2; 1 > 3
Emotional care	3.38 (1.65)	1.68 (1.98)	2.20 (1.90)	2.51 (1.61)	2.52 (1.95)	15.39 (4)	**	1 > 2
Sibling care	2.26 (2.18)	1.11 (1.66)	1.16 (1.74)	2.26 (2.11)	2.48 (2.02)	16.40 (4)	**	5 > 3

Note. For gender, 16 participants indicating another answer were excluded from analyses. For family living arrangement, 19 participants indicating another arrangement were excluded from analyses. ASR = adjusted standardized residuals. Bold ASR reflects a significant over- or underrepresentation. * *p* < 0.05. ** *p* < 0.01. *** *p* < 0.001. Cluster 1: Low HRQoL; Cluster 2: High HRQoL; Cluster 3: Moderate HRQoL with High Social Acceptance; Cluster 4: Moderate HRQoL with Low Social Acceptance; Cluster 5: High Well-Being, High Moods and Emotions, and High Social Support and Peers. Kruskal-Wallis tests were performed for perceived academic performance, illness perception, and caring activities. For post-hoc tests, pairwise comparisons using Mann-Whitney tests (with Bonferroni corrections) were conducted.

## Data Availability

The datasets used during the current study are available from the corresponding author on reasonable request.
